# Clinical text classification with rule-based features and knowledge-guided convolutional neural networks

**DOI:** 10.1186/s12911-019-0781-4

**Published:** 2019-04-04

**Authors:** Liang Yao, Chengsheng Mao, Yuan Luo

**Affiliations:** 1 Northwestern University, Chicago 60611, IL USA; 2 Department of Preventive Medicine, Feinberg School of Medicine, Northwestern University, Chicago 60611, IL USA

**Keywords:** Clinical text classification, Obesity challenge, Convolutional neural networks, Word embeddings, Entity embeddings

## Abstract

**Background:**

Clinical text classification is an fundamental problem in medical natural language processing. Existing studies have cocnventionally focused on rules or knowledge sources-based feature engineering, but only a limited number of studies have exploited effective representation learning capability of deep learning methods.

**Methods:**

In this study, we propose a new approach which combines rule-based features and knowledge-guided deep learning models for effective disease classification. Critical Steps of our method include recognizing trigger phrases, predicting classes with very few examples using trigger phrases and training a convolutional neural network (CNN) with word embeddings and Unified Medical Language System (UMLS) entity embeddings.

**Results:**

We evaluated our method on the 2008 Integrating Informatics with Biology and the Bedside (i2b2) obesity challenge. The results demonstrate that our method outperforms the state-of-the-art methods.

**Conclusion:**

We showed that CNN model is powerful for learning effective hidden features, and CUIs embeddings are helpful for building clinical text representations. This shows integrating domain knowledge into CNN models is promising.

## Introduction

Clinical records are an important type of electronic health record (EHR) data and often contain detailed and valuable patient information and clinical experiences of doctors. As a basic task of natural language processing, text classification plays an critical role in clinical records retrieval and organization, it can also support clinical decision making and cohort identification [1, 2].

Existing clinical text classification studies often use different forms of knowledge sources or rules for feature engineering [3–7]. But most of the studies could not learn effective features automatically, while deep learning methods have shown powerful feature learning capability recently in the general domain [8].

In this study, we propose a new method which combines rule-based feature engineering and knowledge-guided deep learning techniques for disease classification. We first identify trigger phrases using rules, then use these trigger phrases to predict classes with very few examples, and finally train a convolutional neural network (CNN) on the trigger phrases with word embeddings and Unified Medical Language System (UMLS) [9] Concept Unique Identifiers (CUIs) with entity embeddings. We evaluated our method on the 2008 Integrating Informatics with Biology and the Bedside (i2b2) obesity challenge [10], a multilabel classification task focused on obesity and its 15 most common comorbidities (diseases). The experimental results show that our method outperforms state-of-the-art methods for the challenge.

## Related Work

### Clinical text classification

A systematic literature review of clinical coding and classification systems has been conducted by Stanfill et al. [11]. Some challenge tasks in biomedical text mining also focus on clinical text classification, e.g., Informatics for Integrating Biology and the Bedside (i2b2) hosted text classification tasks on determining smoking status [10], and predicting obesity and its co-morbidities [12]. In this work, we focus on the obesity challenge [12]. Among the top ten systems of obesity challenge, most are rule-based systems, and the top four systems are purely rule-based.

Many approaches for clinical text classification rely on biomedical knowledge sources [3]. A common approach is to first map narrative text to concepts from knowledge sources like Unified Medical Language System (UMLS), then train classifiers on document representations that include UMLS Concept Unique Identifiers (CUIs) as features [6]. More knowledge-intensive approaches enrich the feature set with related concepts [4] for apply semantic kernels that project documents that contain related concepts closer together in a feature space [7]. Similarly, Yao et al. [13] proposed to improve distributed document representations with medical concept descriptions for traditional Chinese medicine clinical records classification.

On the other hand, some clinical text classification studies use various types of information instead of knowledge sources. For instance, effective classifiers have been designed based on regular expression discovery [14] and semi-supervised learning [15, 16]. Active learning [17] has been applied in clinical domain, which leverages unlabeled corpora to improve the classification of clinical text.

Although these methods used rules, knowledge sources or different types of information in many ways. They seldom use effective feature learning methods, while deep learning methods are recently widely used for text classification and have shown powerful feature learning capabilities.

### Deep learning for clinical data mining

Recently, deep learning methods have been successfully applied to clinical data mining. Two representative deep models are convolutional neural networks (CNN) [18, 19] and recurrent neural networks (RNN) [20, 21]. They achieve state of the art performances on a number of clinical data mining tasks. Beaulieu-Jones et al. [22] designed a neural network approach to construct phenotypes for classifying patient disease status. The model performed better than decision trees, random forests and Support Vector Machines (SVM). They also showed to successfully learn the structure of high-dimensional EHR data for phenotype stratification. Gehrmann et al. [23] compared CNN to the traditional rule-based entity extraction systems using the cTAKES and Logistic Regression (LR) with n-gram features. They tested ten different phenotyping tasks on discharge summaries. CNN outperformed other phenotyping algorithms on the prediction of the ten phenotypes, and they concluded that deep learning-based NLP methods improved the patient phenotyping performance compared to other methods. Luo et al. applied both CNN, RNN, and Graph Convolutional Networks (GCN) to classify the semantic relations between medical concepts in discharge summaries from the i2b2-VA challenge dataset [24] and showed that CNN, RNN and GCN with only word embedding features can obtain similar or better performances compared to state-of-the-art systems by challenge participants with heavy feature engineering [25–27]. Wu et al. [28] applied CNN using pre-trained embeddings on clinical text for named entity recognization. They showed that their models outperformed the conditional random fields (CRF) baseline. Geraci et al. [29] applied deep learning models to identify youth depression in unstructured text notes. They obtained a sensitivity of 93.5% and a specificity of 68%. Jagannatha et al. [30, 31] experimented with RNN, long short-term memory (LSTM), gated recurrent units (GRU), bidirectional LSTM, combinations of LSTM with CRF, to extract clinical concepts from texts. They demonstrated that all RNN variants outperformed the CRF baseline. Lipton et al. [32] evaluated LSTM in phenotype prediction using multivariate time series clinical measurements. They showed that their model outperformed multi-layer perceptron (MLP) and LR. They also concluded that combining MLP and LSTM leads to the best performance. Che et al. [33] also applied deep neural networks to model time series in ICU data. They introduced a Laplacian regularization process on the sigmoid layer based on medical knowledge bases and other structured knowledge. In addition, they designed an incremental training procedure to iteratively add neurons to the hidden layer. They then used causal inference to analyze and interpret hidden layer representations. They showed that their method improved the performance of phenotype identification, the model also converges faster and has better interpretation.

Although deep learning techniques have been well studied in clinical data mining, most of these works do not focus on long clinical text classification (e.g., an entire clinical note) or utilize knowledge sources, while we propose a novel knowledge-guided deep learning method for clinical text classification.

## Obesity challenge

The objective of the i2b2 2008 obesity challenge [12] is to assess text classification methods for determining patient disease status with respect to obesity and 15 of its comorbidities: Diabetes mellitus (DM), Hypercholesterolemia, Hypertriglyceridemia, Hypertension, atherosclerotic cardiovascular disease (CAD), Heart failure (CHF), Peripheral vascular disease (PVD), Venous insufficiency, Osteoarthritis (OA), Obstructive sleep apnea (OSA), Asthma, Gastroesophageal reflux disease (GERD), Gallstones, Depression, and Gout. Our goal is to label each document as either Present (Y), Absent (N), Questionable (Q) or Unmentioned (U) for each disease. Macro *F*_1_ score is the primary metric for evaluating and ranking classification methods.

The challenge consists of two tasks, namely textual task and intuitive task. The textual task is to identify explicit evidences of the diseases, while the intuitive task focused on the prediction of the disease status when the evidence is not explicitly mentioned. Thus, the Unmentioned (U) class label was excluded from the intuitive task. The classes are distributed very unevenly: there are only few N and Q examples in textual task data set and few Q examples in intuitive task data set, as shown in Table 1. There exist classes even without training example. For instance, there is no training example with Q and N label for Depression in textual task, and there is no training example with Q label for Gallstones in intuitive task. The details of the datasets can be found in [12].

**Table 1:** The class distribution in the obesity challenge datasets

Label	Training Set		Test Set	
	Textual	Intuitive	Textual	Intuitive
Y	3208	3267	2192	2285
N	87	7362	65	5100
Q	39	26	17	14
U	8296	0	5770	0

## Method

Our method contains three steps: (1). identifying trigger phrases; (2). predicting classes with very few examples using trigger phrases; (3). learning a knowledge-guided CNN for more populated classes. Our implementation is available at https://github.com/yao8839836/obesity. We use Solt’s system [5] to recognize trigger phrases and predict classes with very few examples. Solt’s system is a very powerful rule-based system. It ranked the first in the intuitive task and the second in the textual task and overall the first in the obesity challenge. Solt’s system can identify very informative trigger phrases with different contexts (positive, negative or uncertain). We use the Perl implementation: https://github.com/yao8839836/obesity/tree/master/perl_classifier of Solt’s system provided by the authors.

### Trigger phrases identification

We recognize trigger phrases following Solt’s system [5]. We first conduct the same preprocessing like abbreviation resolution and family history removing. We then use the disease names (class names), their directly associated terms and negative/uncertain words to recognize trigger phrases. The trigger phrases are disease names (e.g., Gallstones) and their alternative names (e.g., Cholelithiasis) with/without negative or uncertain words.

### Predicting classes with very few examples using trigger phrases

As the classes in obesity challenge are very unbalanced, and some classes even don’t have training examples, we could not make prediction for these classes using machine learning methods and resort to rules defined in Solt’s system [5]. We exclude classes with very few examples in training set of each disease. Specifically, we remove examples with Q label in intuitive task and remove examples with Q or N label for textual task. Then for examples in test set, we use trigger phrases to predict their labels. As Solt’s system [5], we assume positive trigger phrases (disease names and alternatives without uncertain or negative words) are prior to negative trigger phrases, and negative trigger phrases are prior to uncertain trigger phrases. Therefore, if a clinical record contains uncertain trigger phrases and dosen’t contain positive or negative trigger phrases, we label it as Q. Similarly, if a clinical record contains negative trigger phrases and dosen’t contain positive trigger phrases, we label it as N.

### Knowledge-guided convolutional neural networks

**Fig. 1:**
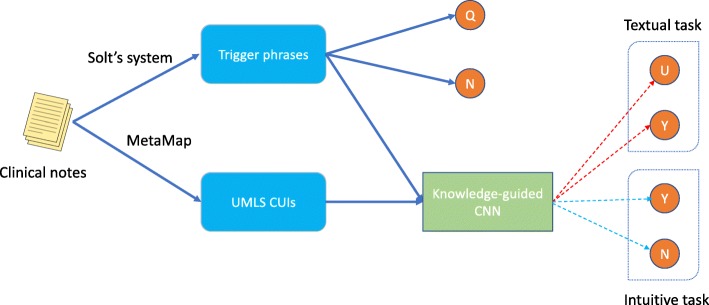
The test phase of our method

After excluding classes with very few examples, only two classes remain in the training set of each disease (Y and N for intuitive task, Y and U for textual task). We learn a CNN on positive trigger phrases and UMLS CUIs in training records, then classify test examples using the trained CNN model. CNN is a powerful deep learning model for text classification, and it performs better than recurrent neural networks in our preliminary experiment. The test phase of our method is given in Fig. 1. If a record in test set is labeled Q or N by Solt’s system, we trust Solt’s system. Otherwise, we use the CNN to predict the label of the record.

For each disease, we feed its positive trigger phrases with word2vec [34] word embeddings to CNN. We employed the 200 dimensional pre-trained word embeddings learned from MIMIC-III [35] clinical notes. We experimented with 100, 200, 300, 400, 500 and 600 dimensional word embeddings, and found using 200 dimensional word embeddings achieves the best performance.

We also utilize medical knowledge base to enrich the CNN model input. We link the full clinical text to CUIs in UMLS [9] via MetaMap [36]. Each clinical record is represented as a bag of CUIs after entity linking. We feed 13 types of CUIs which are closely connected to diseases as the input entities of CNN: Body Part, Organ, or Organ Component (T023), Finding (T033), Laboratory or Test Result (T034), Disease or Syndrome (T047), Mental or Behavioral Dysfunction (T048), Cell or Molecular Dysfunction (T049), Laboratory Procedure (T059), Diagnostic Procedure (T060), Therapeutic or Preventive Procedure (T061), Pharmacologic Substance (T121), Biomedical or Dental Material (T122), Biologically Active Substance (T123) and Sign or Symptom (T184). We list these CUIs types with type unique identifier (TUI) in Table 2. We found using the subset of CUIs achieves better performances than using all CUIs. We employ pre-trained CUIs embeddings made by [37] as the input entity representations of CNN.

**Table 2:** The types of CUIs we used

TUI	Semantic type description
T023	Body Part, Organ, or Organ Component
T033	Finding
T034	Laboratory or Test Result
T047	Disease or Syndrome
T048	Mental or Behavioral Dysfunction
T049	Cell or Molecular Dysfunctions
T059	Laboratory Procedure
T060	Diagnostic Procedure
T061	Therapeutic or Preventive Procedure
T121	Pharmacologic Substance
T122	Biomedical or Dental Material
T123	Biologically Active Substance
T184	Sign or Symptom

**Fig. 2:**
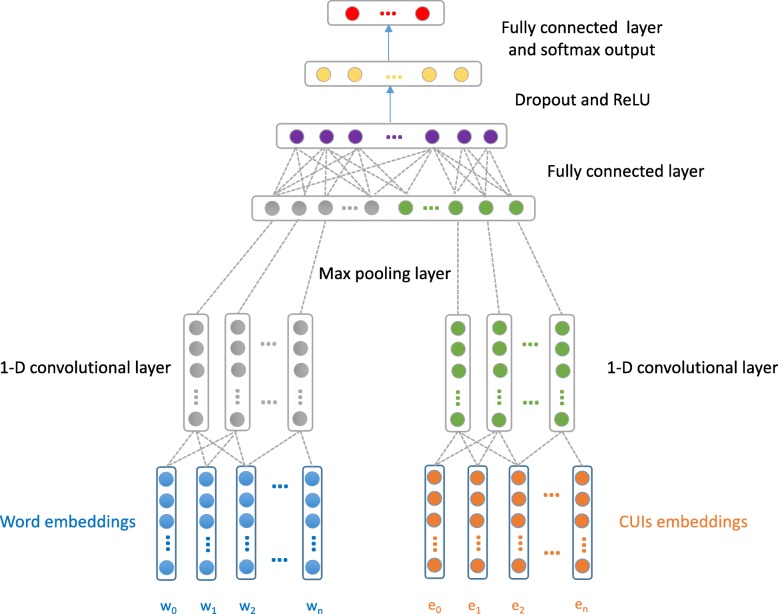
Our knowledge-guided convolutional neural network architecture

Our CNN architecture is given in Fig. 2. The input layer looks up word embeddings of positive trigger phrases and entity embeddings of selected CUIs in each clinical record. *w*_0_,*w*_1_,*w*_2_,…,*w*_*n*_ are words in positive trigger phrases and *e*_0_,*e*_1_,*e*_2_,…,*e*_*n*_ are CUIs in a record. A one dimensional convolution layer is built on the word embeddings and entity embeddings. We use max pooling to select the most prominent feature with the highest value in the convolutional feature map, then concatenate the max pooling results of word embeddings and entity embeddings. The concatenated hidden representations are fed into a fully-connected layer, then a dropout and a ReLU activation layer. Lastly, a fully-connected layer is fed to a softmax layer, whose output is the multinomial distribution over labels.

We implement our knowledge-guided CNN model using TensorFlow [38], a popular deep learning framework. We set the following parameters for our CNN model: the convolution kernel size: 5, the number of convolution filters: 256, the dimension of hidden layer in the fully connected layer: 128, dropout keep probability: 0.8, the number of learning epochs: 30, batch size: 64, learning rate: 0.001. We also experimented with other settings of the parameters but didn’t find much difference. We use softmax cross entropy loss and Adam optimizer [39].

## Results

Tables 3 and 4 show Macro *F*_1_ scores and Micro *F*_1_ scores of our method and Solt’s system. We report results of both the Solt’s paper [5] and the Perl implementation because we base our method on the Perl implementation and we found there are some differences between the paper’s results and Perl implementation’s results. This is likely due to further feature engineering that are not reflected when Solt et al. submitted classification output to the challenge. For completeness of the results, we show the performances from both Solt’s paper and code. We also report the results of our method when using only word embeddings as CNN input.

**Table 3:** Macro *F*_1_ scores and Micro *F*_1_ scores of Solt’s system [5] (paper) and our method with word and entity embeddings

Disease	Solt’s paper [5]				Our method with word & entity embeddings			
	Textual		Intuitive		Textual		Intuitive	
	Macro *F*_1_	Micro *F*_1_	Macro *F*_1_	Micro *F*_1_	Macro *F*_1_	Micro *F*_1_	Macro *F*_1_	Micro *F*_1_
Asthma	0.9434	0.9921	0.9784	0.9894	0.9434	0.9921	0.9784	0.9894
CAD	0.8561	0.9256	0.6122	0.9192	0.8551	0.9235	**0.6233**	**0.9345**
CHF	0.7939	0.9355	0.6236	0.9315	0.7939	0.9355	0.6236	0.9315
Depression	0.9716	0.9842	0.9346	0.9539	0.9716	0.9842	**0.9602**	**0.9727**
DM	0.9032	0.9761	0.9682	0.9729	0.9056	0.9801	0.9731	0.9770
Gallstones	0.8141	0.9822	0.9729	0.9857	0.8141	0.9822	0.9689	0.9837
GERD	0.4880	0.9881	0.5768	0.9131	0.4880	0.9881	0.5768	0.9131
Gout	0.9733	0.9881	0.9771	0.9900	0.9733	0.9881	0.9771	0.9900
Hypercholesterolemia	0.7922	0.9721	0.9053	0.9072	0.7922	0.9721	**0.9113**	0.9118
Hypertension	0.8378	0.9621	0.8851	0.9283	0.8378	0.9621	**0.9240**	**0.9484**
Hypertriglyceridemia	0.9732	0.9980	0.7981	0.9712	0.9434	0.9961	0.7092	0.9630
OA	0.9594	0.9761	0.6286	0.9589	0.9626	0.9781	0.6307	0.9610
Obesity	0.4879	0.9675	0.9724	0.9732	0.4885	0.9696	0.9747	0.9754
OSA	0.8781	0.9920	0.8805	0.9939	0.8781	0.9920	0.8805	0.9939
PVD	0.9682	0.9862	0.6348	0.9763	0.9682	0.9862	0.6314	0.9742
Venous insufficiency	0.8403	0.9822	0.8083	0.9625	**0.8816**	**0.9882**	0.8083	0.9625
Overall	0.8000	0.9756	0.6745	0.9590	**0.8016**	**0.9763**	**0.6768**	**0.9624**

Scores in bold font means they are higher than the corresponding scores of the paper and Perl implementation

**Table 4:** Macro *F*_1_ scores and Micro *F*_1_ scores of Solt’s system [5] (code) and our method with word embeddings only

Disease	Solt’s code				Our method with word embeddings only			
	Textual		Intuitive		Textual		Intuitive	
	Macro *F*_1_	Micro *F*_1_	Macro *F*_1_	Micro *F*_1_	Macro *F*_1_	Micro *F*_1_	Macro *F*_1_	Micro *F*_1_
Asthma	0.9434	0.9921	0.9784	0.9894	0.9434	0.9921	0.9784	0.9894
CAD	0.8551	0.9235	0.6122	0.9192	0.8551	0.9235	0.6122	0.9192
CHF	0.7939	0.9355	0.6236	0.9315	0.7939	0.9355	0.6236	0.9315
Depression	0.9716	0.9842	0.9346	0.9539	0.9716	0.9842	**0.9602**	**0.9767**
DM	0.9056	0.9801	0.9731	0.9770	0.9056	0.9801	0.9731	0.9770
Gallstones	0.8141	0.9822	0.9729	0.9857	0.8141	0.9822	0.9729	0.9857
GERD	0.4880	0.9881	0.5768	0.9131	0.4880	0.9881	0.5768	0.9131
Gout	0.9733	0.9881	0.9771	0.9900	0.9733	0.9881	0.9771	0.9900
Hypercholesterolemia	0.7922	0.9721	0.9101	0.9118	0.7922	0.9721	0.9042	0.9049
Hypertension	0.8378	0.9621	0.8861	0.9283	0.8378	0.9621	**0.9240**	**0.9484**
Hypertriglyceridemia	0.9732	0.9980	0.7092	0.9630	0.9732	0.9980	0.7092	0.9630
OA	0.9626	0.9781	0.6307	0.9610	0.9626	0.9781	0.6307	0.9610
Obesity	0.4885	0.9696	0.9747	0.9754	0.4885	0.9696	0.9747	0.9754
OSA	0.8781	0.9920	0.8805	0.9939	0.8781	0.9920	0.8805	0.9939
PVD	0.9682	0.9862	0.6314	0.9742	0.9682	0.9862	0.6314	0.9742
Venous insufficiency	0.8403	0.9822	0.8083	0.9625	0.8403	0.9822	0.8083	0.9625
Overall	0.8014	0.9760	0.6745	0.9592	0.8014	0.9760	**0.6760**	**0.9612**

Scores in bold font means they are higher than the corresponding scores of the paper and Perl implementation

From the two tables, we can note that the Perl implementation performs slightly better than the paper, the authors might not submit their best results to the obesity challenge. We can also see that CNN model with word embeddings only performs better than the Perl implementation in intuitive task, which means using a deep learning model can learn effective features for better classification. The input trigger phrases for CNN are the same as the trigger phrases for Y/U (textual task) or Y/N (intuitive task) labeling in the Perl code. The results in the textual task are not improved when using word embeddings only, because the textual task needs explicit evidences to label the records, and the positive trigger phrases contain enough information, therefore CNN with word embeddings only may not be particularly helpful. Nevertheless, after adding CUIs embeddings as additional input, more scores for different diseases are improved, and the overall *F*_1_ scores are higher than Solt’s system in the two tasks. This is likely due to the fact that the disambiguated CUIs are closely connected to diseases and their embeddings have more semantic information, which is beneficial for disease classification. To the best of our knowledge, we have achieved the highest overall *F*_1_ scores in intuitive task so far.

Note that the *F*_1_ scores of Solt’s paper and Perl implementation remain the same, while our model produces slightly different *F*_1_ scores in different runs. We run our model 10 times and observed that the overall Macro *F*_1_ scores and Micro *F*_1_ scores are significantly higher than Solt’s paper and implementation (*p* value <0.05 based on student *t* test). We checked the cases our method failed to predict correctly. and found the most error cases are caused by using Solt’s positive trigger phrases. For many error cases, our method predicted N or U when no positive trigger phrases are identified, but the real labels are Y. For some other cases, our method predicted Y when positive trigger phrases are identified, but the real labels are N or U. For some diseases, our proposed method and Solt’s system achieved a very high Micro *F*_1_ but a low Macro *F*_1_. This is due to the fact that there are only a few Q or N records for these diseases (i.e., imbalanced class ratio), and we could not identify effective negative/uncertain trigger phrases using Solt’s rules. The regular expressions in Solt’s system can be further enriched so that we can identify trigger phrases more accurately.

We also compared our method with two commonly used classifiers: Logistic Regression and linear kernel support Vector Machine (SVM). We use LogisticRegression and LinearSVC class in scikit-learn as our implementations. For fair comparison, we use the same training set as knowledge-guided CNN. We represent a record as a binary vector, each dimension means whether an unique word is in its positive trigger phrases. For test examples, we also use Solt’s system to predict Q and N. If a test example is not labeled Q or N by Solt’s system, we use Logistic Regression or SVM to predict the label. Table 5 shows the results, we can observe that the results are similar to our method with word embeddings only, which means positive trigger phrases themselves are informative enough, while word embeddings could not help to improve the performances. Nevertheless, we run our model 10 times and observed that the overall Macro *F*_1_ scores and Micro *F*_1_ scores are significantly higher than SVM and Logistic Regression (*p* value <0.05 based on student *t* test), which verifies the effectiveness of CUIs embeddings again.

**Table 5:** Macro *F*_1_ scores and Micro *F*_1_ scores of Logistic Regression and SVM

Disease	Logistic Regression				SVM			
	Textual		Intuitive		Textual		Intuitive	
	Macro *F*_1_	Micro *F*_1_	Macro *F*_1_	Micro *F*_1_	Macro *F*_1_	Micro *F*_1_	Macro *F*_1_	Micro *F*_1_
Asthma	0.9434	0.9921	0.9784	0.9894	0.9434	0.9921	0.9784	0.9894
CAD	0.8551	0.9235	0.6204	0.9301	0.8551	0.9235	0.6122	0.9192
CHF	0.7939	0.9355	0.6236	0.9315	0.7939	0.9355	0.6236	0.9315
Depression	0.9716	0.9842	0.9573	0.9706	0.9716	0.9842	0.9573	0.9706
DM	0.9056	0.9801	0.9731	0.9770	0.9056	0.9801	0.9731	0.9770
Gallstones	0.8141	0.9822	0.9729	0.9857	0.8141	0.9822	0.9729	0.9857
GERD	0.4880	0.9881	0.5768	0.9131	0.4880	0.9881	0.5768	0.9131
Gout	0.9733	0.9881	0.9771	0.9900	0.9733	0.9881	0.9771	0.99
Hypercholesterolemia	0.7922	0.9721	0.9043	0.9049	0.7922	0.9721	0.9134	0.9142
Hypertension	0.8378	0.9621	0.9271	0.9507	0.8378	0.9621	0.9271	0.9507
Hypertriglyceridemia	0.9732	0.9980	0.7092	0.9630	0.9732	0.9980	0.7092	0.9630
OA	0.9626	0.9781	0.6307	0.961	0.9626	0.9781	0.6307	0.9610
Obesity	0.4885	0.9696	0.9747	0.9754	0.4885	0.9696	0.9747	0.9754
OSA	0.8781	0.992	0.8805	0.9939	0.8781	0.9920	0.8805	0.9939
PVD	0.9682	0.9862	0.6314	0.9742	0.9682	0.9862	0.6314	0.9742
Venous insufficiency	0.8403	0.9822	0.8083	0.9625	0.8403	0.9822	0.8083	0.9625
Overall	0.8014	0.9760	0.6764	0.9619	0.8014	0.9760	0.6764	0.9618

Classes with very few examples are labeled by Solt’s system

## Discussion

We note that the knowledge features part does not improve much. In fact, we think MetaMap will indeed introduce some noisy and unrelated CUIs, as previous studies also showed. To remedy this, following Weng et al. [40], we only kept CUIs from selected semantic types that are considered most relevant to clinical tasks. We found that filtering CUIs based on semantic types did lead to moderate performance improvement over using all CUIs. In another related computational phenotyping study [41], we found that manually curated CUI set resulted in significant performance improvement. We believe that improving entity recognition and integrating word/entity sense disambiguation will improve the performance, and plan to explore such directions in future work.

## Conclusion

In this work, we propose a novel clinical text classification method which combines rule-based feature engineering and knowledge-guided deep learning. Specifically, we use rules to identify trigger phrases which contain diseases names, their alternative names and negative or uncertain words, then use these trigger phrases to predict classes with very limited examples, and finally train a knowledge-guided CNN model with word embeddings and UMLS CUIs entity embeddings. The evaluation results on the obesity challenge demonstrate that our method outperforms state-of-the-art methods for the challenge. We showed that CNN model is powerful for learning effective hidden features, and CUIs embeddings are helpful for building clinical text representations. This shows integrating domain knowledge into CNN models is promising. In our future work, We plan to design more principled methods and evaluate our methods on more clinical text datasets.
